# International School-Based HPV Vaccination Programmes: A Comparative Analysis and Recommendations for Adaptation to the French Context

**DOI:** 10.3390/vaccines14100873

**Published:** 2026-10-02

**Authors:** Sarah Soupey, Laure Hermet

**Affiliations:** 1 Department of Public Health, Nice University Hospital, 06000 Nice, France; soupey.s@chu-nice.fr; 2 UR2CA–RESPECT, Université Côte d’Azur, 06000 Nice, France

**Keywords:** HPV vaccination, school-based vaccination, implementation strategies, contextual adaptation, transferability, public health

## Abstract

Background/Objectives: School-based vaccination is a major strategy for improving human papillomavirus (HPV) vaccination coverage, but implementation varies across countries. This study aimed to identify common organisational components of international school-based HPV vaccination programmes, compare their implementation, and examine their transferability to France. Methods: A comparative documentary analysis of programmes in four purposively selected countries (Australia, England, Finland, and Sweden) was conducted between July and August 2026. Scientific and institutional sources were analysed using a common framework covering programme organisation, consent, communication, catch-up, equity, and monitoring. The identified components were examined in relation to the French organisational, regulatory, and sociocultural context. Results: Six common organisational components were identified: national leadership and structured local implementation; school-based delivery; organised consent; multichannel communication tailored to families and adolescents; catch-up mechanisms; and information systems supporting identification and follow-up of the target population. Implementation approaches varied across countries, suggesting that transferability depends more on the functions fulfilled by these components than on replicating specific organisational arrangements. Conclusions: No single school-based HPV vaccination model can be directly transferred across countries. Adapting the identified components to France could support a continuous, coordinated vaccination pathway and reduce missed vaccination opportunities while accounting for national specificities.

## 1. Introduction

Human papillomavirus (HPV) vaccination represents one of the most effective public health interventions for preventing HPV-related cancers, particularly cervical cancer [1,2]. To support the elimination of cervical cancer as a public health problem, the World Health Organization (WHO) has set a target of achieving 90% HPV vaccination coverage among girls before the age of 15 years [3]. To contribute to this target, many countries have implemented school-based vaccination programmes, which have demonstrated their effectiveness in achieving high vaccination coverage and substantially reducing the incidence of HPV infections and precancerous lesions [4,5,6].

Despite converging international recommendations and strong evidence regarding the safety, effectiveness, and cost-effectiveness of HPV vaccination [1,7], vaccination coverage remains highly heterogeneous worldwide. While some countries, such as Australia and Sweden, now achieve coverage rates exceeding 80% [8,9,10,11], others, including France, continue to fall short of established targets [12]. These differences cannot be explained solely by vaccine characteristics or national recommendations. They also reflect differences in programme implementation, health policies, the organisation of health and education systems, communication strategies, and the sociocultural contexts in which these interventions are delivered [4,13,14].

This heterogeneity raises a fundamental question for public health decision-makers. While the strategies implemented in countries that have achieved the highest vaccination coverage are now well documented, how can they be transferred to another national context without compromising their effectiveness? In other words, which essential components should be preserved, and which implementation approaches should be adapted to local specificities to ensure the effectiveness of a school-based vaccination programme? The existing literature has extensively examined determinants of HPV vaccine acceptance and the implementation or evaluation of individual national programmes [4,14]. However, the question of how organisational components of successful school-based programmes can be transferred and adapted across national contexts remains less explored.

France provides a particularly relevant case study for addressing this issue. Since 2023, a national school-based HPV vaccination campaign has been implemented to improve vaccination coverage among adolescents [12,15]. This initiative represents a major development in French vaccination policy. However, its implementation takes place within a specific organisational, regulatory, cultural, and societal context that differs from those of countries that have achieved the highest vaccination coverage. The challenge is therefore not to replicate a foreign model exactly, but rather to identify the mechanisms contributing to its success, determine the extent to which they are transferable, and assess how they can be adapted to the French context.

The objective of this study was to analyse international school-based HPV vaccination programmes achieving high vaccination coverage in order to identify common organisational components, explore their transferability, and propose recommendations for adaptation to the French context.

## 2. Materials and Methods

### 2.1. Study Design

**Figure 1:**
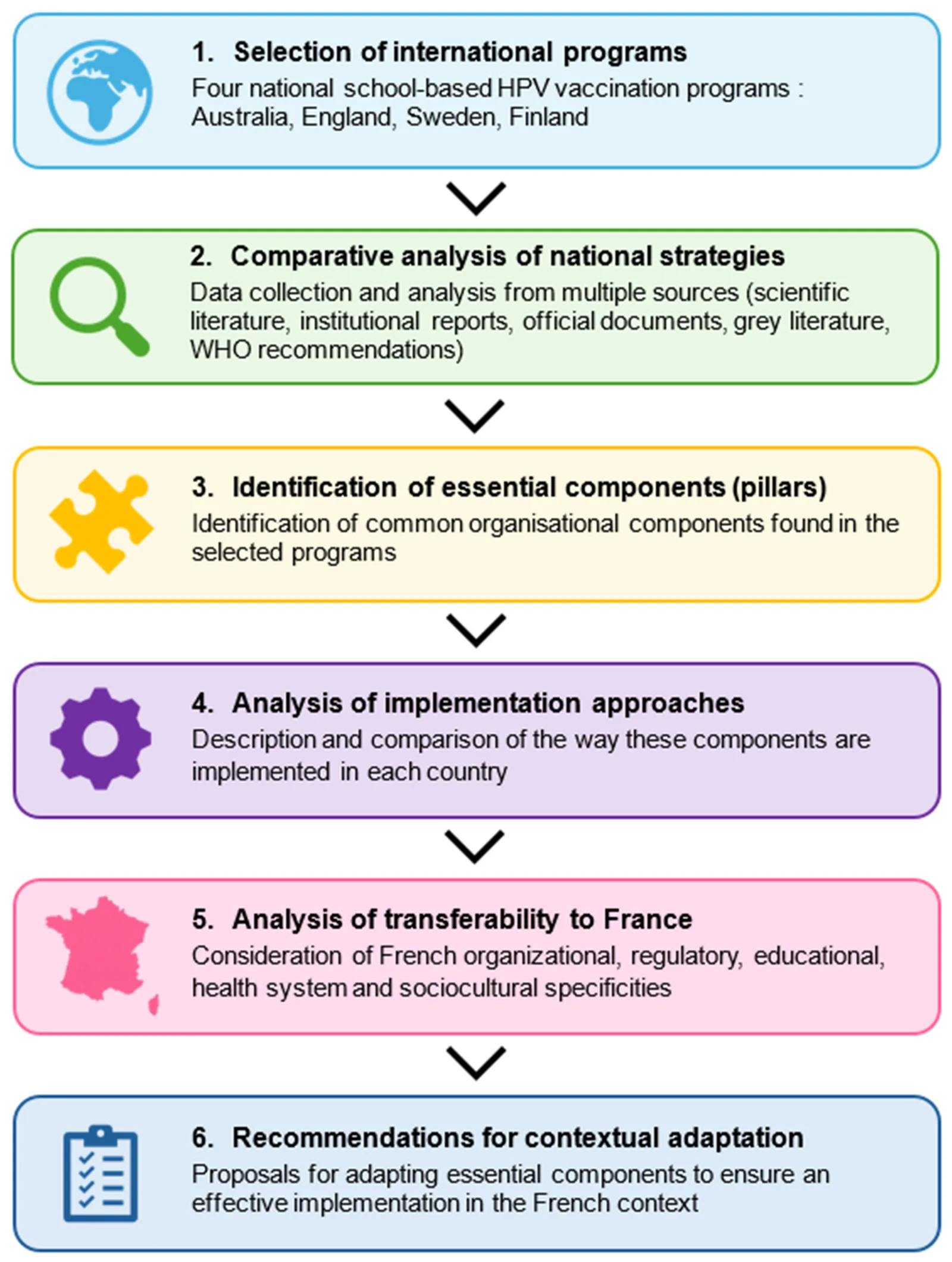
Comparative analysis and contextual adaptation process for international school-based HPV vaccination programmes.

This study was based on a comparative analysis of national school-based human papillomavirus (HPV) vaccination strategies implemented in several countries with established school-based vaccination programmes. The objective was not to assess the clinical effectiveness of HPV vaccines, but to identify the common organisational components identified across these programmes, compare their implementation approaches in different national contexts, and explore their transferability in order to formulate recommendations for adaptation to the French context. The analytical process, from the selection of international programmes to the development of recommendations for contextual adaptation in France, is presented in Figure 1.

### 2.2. Country Selection

Country selection was conducted in several stages. An initial PubMed search was performed to identify countries with school-based HPV vaccination programmes and to document reported vaccination coverage among adolescents. The initial selection criterion was based on the World Health Organization (WHO) target of achieving 90% HPV vaccination coverage among girls before the age of 15 years [3]. However, applying a 90% threshold would have resulted in a very limited number of countries meeting both the vaccination coverage criterion and the other predefined selection criteria. A similar limitation was observed when the threshold was lowered to 80%, with only a small number of eligible countries and insufficient geographical and organisational diversity for the intended comparative analysis. Therefore, a threshold of at least 70% was adopted as a pragmatic criterion to allow the inclusion of countries reporting relatively high vaccination coverage while ensuring sufficient diversity of national contexts and programme organisation. Programmes reporting a minimum coverage of 70% for receipt of one or more HPV vaccine doses among both girls and boys in the reported age group or birth cohort were considered to have sufficiently high coverage to be eligible for inclusion in the analysis. This threshold was used as a pragmatic selection criterion to focus the comparison on programmes reporting relatively high vaccination coverage. The most recent relevant estimates available at the time of the documentary search were used. As the estimates concerned different years and age groups or birth cohorts, they were used to assess eligibility against the selection threshold rather than to compare programme performance directly. Coverage among both girls and boys was selected as the common indicator because HPV vaccination programmes for boys were introduced later in several countries. The study was designed as a purposive comparative analysis rather than a systematic review, the selection was therefore not intended to identify or include all countries implementing school-based HPV vaccination programmes.

For programmes meeting the coverage criterion, a complementary documentary search was conducted to determine whether sufficient information was available to complete the comparative analytical framework. This assessment considered the availability of scientific publications and institutional documents describing programme governance, organisation, implementation and the other dimensions included in the analytical framework (Supplementary Table S1). The documentary search drew on the scientific literature, websites of national health authorities and ministries, and institutional platforms dedicated to vaccination.

The final selection also sought geographical and organisational diversity, rather than including multiple programmes with largely similar delivery models. Australia was retained as an established reference programme in school-based HPV vaccination. Other programmes were considered but not selected for the final comparison. Belgium and Canada were not retained because differences in programme organisation across communities, provinces or territories complicated their analysis as single national cases within the comparative framework. Norway and other UK nations were also considered; however, the selection prioritised diversity over the inclusion of additional programmes with organisational approaches similar to those already represented.

On this basis, four programmes were purposively selected for the comparative analysis: Australia, England, Finland, and Sweden. These programmes allowed comparison of different approaches to organising school-based vaccination depending on the national context, on dedicated vaccination teams, integrated school health services, or decentralised organisational models involving different institutional levels. The selected programmes were intended to provide contrasting, sufficiently documented cases for analysis and to inform consideration of potential adaptations to the French context, rather than to constitute a representative sample of international programmes.

**Figure 2:**
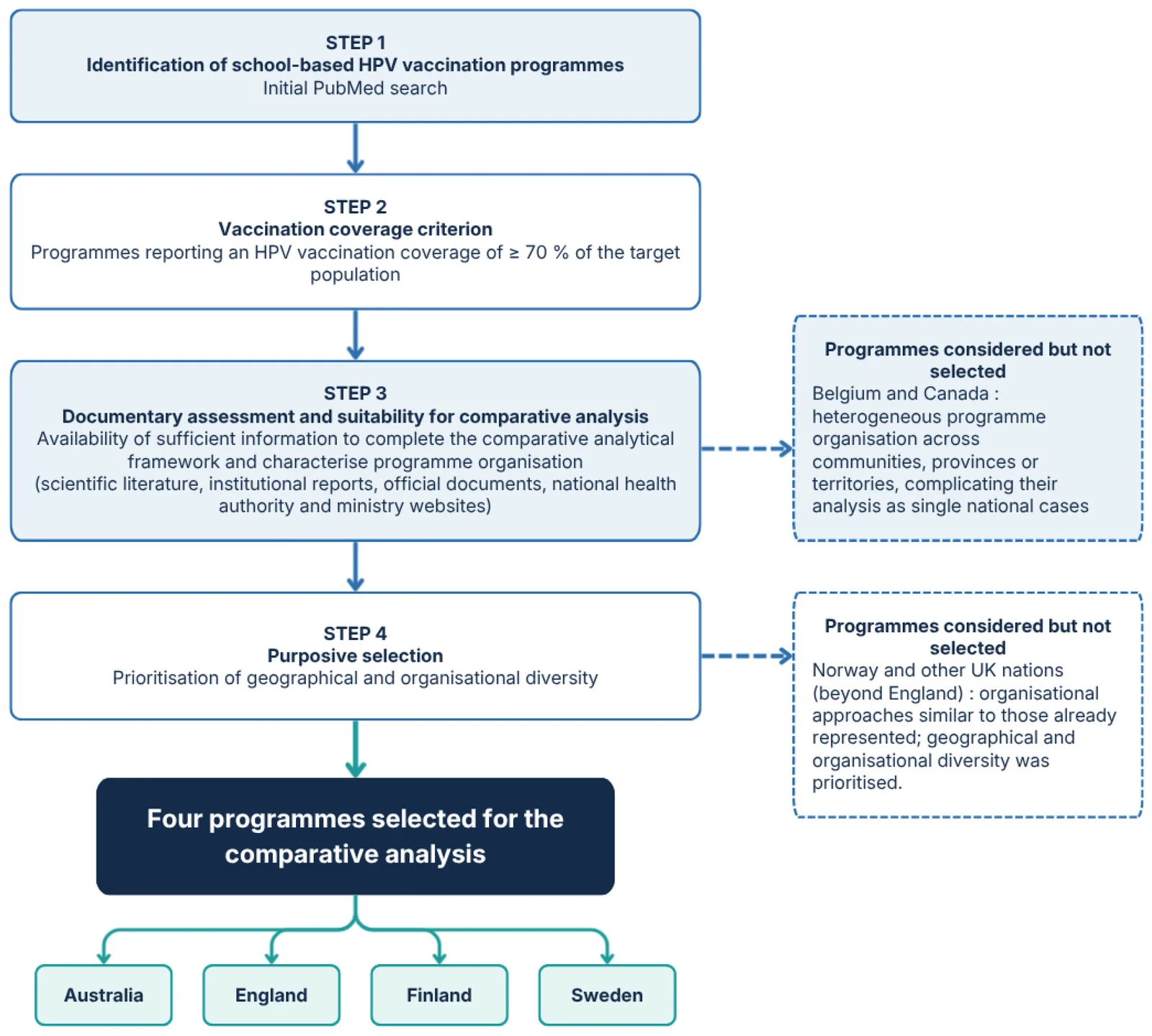
Flow diagram of the purposive selection of international school-based HPV vaccination programmes for the comparative analysis.

The programme selection process, including examples of programmes considered but not retained, is summarised in Figure 2.

The vaccination coverage estimates used to apply the selection criterion are presented in Table 1.

Coverage estimates refer to receipt of at least one HPV vaccine dose. The selection threshold of ≥70% was applied to both girls and boys. Reference years and cohorts differ across countries; the estimates should therefore not be interpreted as directly comparable measures of programme performance.

### 2.3. Documentary Sources

A documentary search was conducted between July and August 2026 to identify countries with school-based HPV vaccination programmes and high levels of vaccination coverage. An initial search was conducted in PubMed using the search terms “HPV vaccination”, “HPV vaccination campaign”, “school-based HPV vaccination” and “vaccination coverage”. This search was used to identify countries with established school-based programmes and to document their vaccination coverage levels.

For each selected country, a targeted documentary search was subsequently conducted to characterise the organisation and implementation of the programme. This involved identifying the official websites of the relevant national government and health authorities, searching these websites for information on HPV vaccination, and identifying any dedicated official website or webpage for the national vaccination programme. Priority was given to national institutional sources, including websites of ministries, health authorities, and organisations responsible for vaccination programmes, as well as, where available, websites specifically dedicated to national HPV vaccination programmes. This information was supplemented, where necessary, by the scientific literature and international recommendations. The scientific and institutional sources used to characterise the four selected programmes are listed in Supplementary Table S1. Publication dates were checked to ensure that the information used reflected the programme arrangements relevant to the period studied.

Documents were retained when they provided information relevant to one or more dimensions of the comparative analytical framework, such as programme organisation, consent procedures, communication strategies, catch-up mechanisms or monitoring systems. The documentary search was targeted rather than systematic, and no formal quality appraisal of the sources was undertaken.

For France, the analysis also drew on official documents relating to the national school-based HPV vaccination campaign implemented since 2023.

### 2.4. Analytical Framework

The information extracted for each country was organised according to the same analytical dimensions to enable cross-country comparison of the programmes. The analysis aimed to identify organisational components common to the programmes studied, compare their implementation approaches across different national contexts, and examine their transferability to the French context (Supplementary Table S1). The dimensions analysed included programme governance and leadership; organisation of school-based vaccination; funding arrangements; target populations; consent procedures; the roles of healthcare professionals and schools; communication strategies targeting adolescents and families; catch-up mechanisms; actions aimed at improving equity of access; and information, monitoring, and evaluation systems. These analytical dimensions were defined before the comparative analysis and used to structure an Excel-based comparative matrix (Microsoft Corporation, Redmond, WA, USA). A comparative synthesis of the matrix was then conducted by examining the information collected for each analytical dimension, identifying recurring organisational and implementation features across the programmes, and grouping these features into common components. No formal qualitative coding procedure was undertaken.

Each programme was also examined to identify differences in implementation approaches, programme-specific characteristics, and elements that could potentially be transferred to the French context, taking into account its organisational, regulatory, and sociocultural characteristics. The comparative matrix used for this analysis is presented in Supplementary Table S1.

### 2.5. Development of Recommendations

The recommendations proposed for the French context were developed through a cross-analysis of the common organisational components identified across the four programmes, the implementation approaches observed across the different countries, and the organisational, regulatory, cultural, and societal specificities of the French context. The objective was not to replicate an existing model, but to identify evidence-informed components that could be preserved while proposing the contextual adaptations required for their implementation within the French system.

### 2.6. Ethical Considerations and Registration

This study was based exclusively on the analysis of publicly available documents and aggregated data. No participants were recruited, and no individual or personal data were collected or analysed. Therefore, the study did not involve human participants and did not require approval from an ethics committee. The study was conducted in accordance with applicable data protection principles and the General Data Protection Regulation (GDPR; Regulation [EU] 2016/679).

## 3. Results

The comparative analysis of school-based HPV vaccination programmes implemented in Australia, Sweden, England, and Finland identified, despite differences in health and administrative systems, a set of common components that may form the foundation of school-based vaccination programmes. The findings did not allow the observed vaccination coverage levels to be directly attributed to any single component, nor did they establish a causal relationship between programme organisation and performance. However, they enabled the identification of recurring organisational characteristics in countries where school-based HPV vaccination had been institutionalised and the analysis of how these characteristics were implemented across different national contexts. The vaccination coverage estimates and population characteristics of the four selected programmes are presented in Table 1, while their vaccination schedules, consent procedures, delivery arrangements, catch-up strategies, communication approaches and information systems are detailed in Supplementary Table S1.

**Figure 3:**
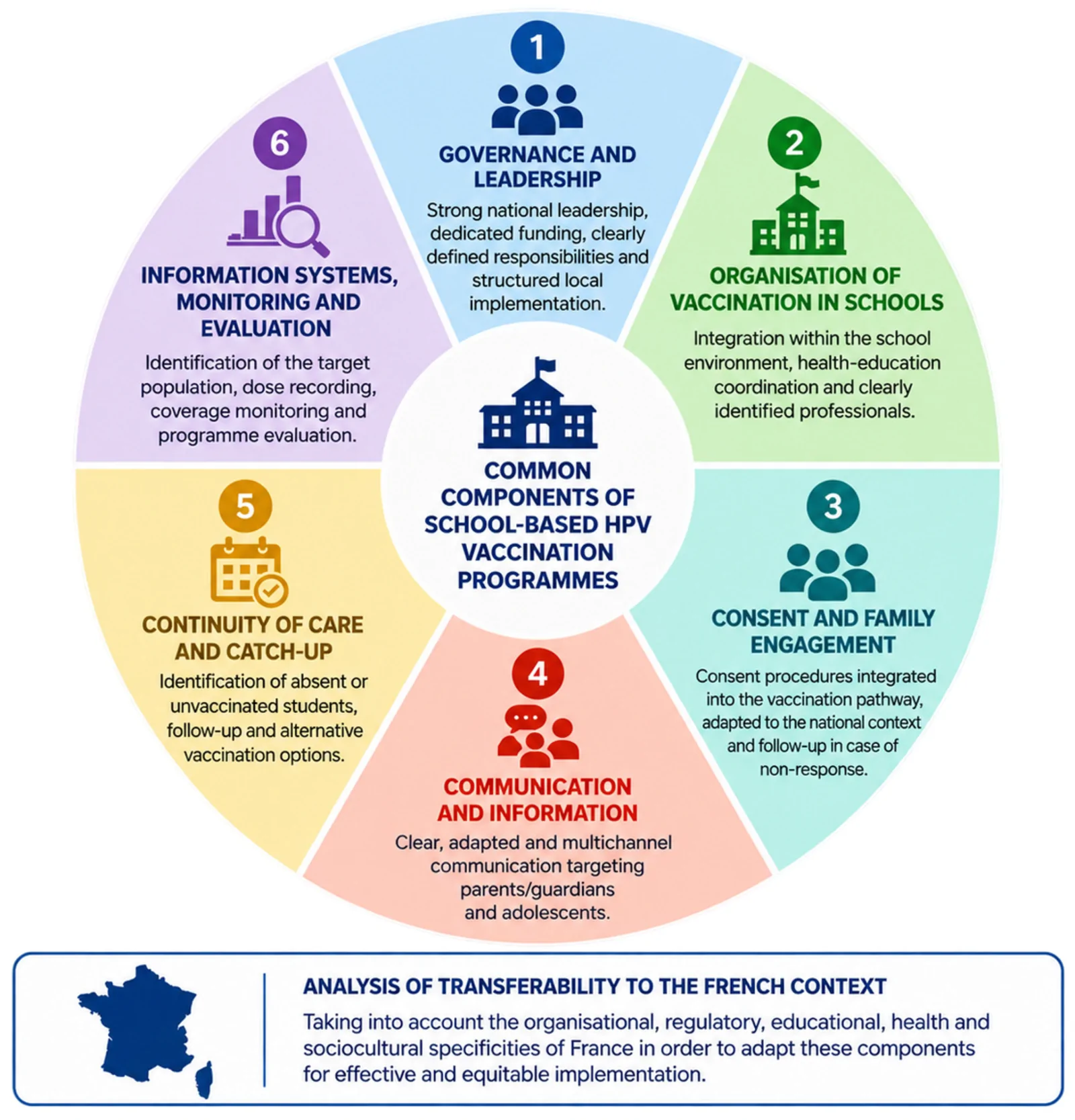
Six common components identified through the comparative analysis of school-based HPV vaccination programmes.

The analysis identified six organisational components common to the programmes studied, although their implementation approaches varied across national contexts (Figure 3).

### 3.1. Strong National Leadership Combined with Structured Local Implementation

The first cross-cutting finding concerned the existence, in all countries studied, of a national framework defining the main parameters of the programme, including the target population, vaccination schedule, and general funding arrangements. Operational implementation, however, relied on different territorial or institutional levels.

Australia had a federal system in which the central government was responsible, in particular, for funding and vaccine procurement, while states and territories contributed to the planning and oversight of programme delivery. In England, the national framework was defined by health authorities and publicly funded, while vaccination was delivered by dedicated School-Aged Immunisation Services (SAIS) teams. In Sweden and Finland, HPV vaccination was more closely integrated into routine school health services, with coordination between national authorities, regional or municipal levels, and school health professionals.

Thus, despite differences in governance, the programmes were based on an explicit allocation of responsibilities across strategic, organisational, and operational levels. National leadership appeared compatible with varying degrees of decentralisation in implementation, provided that the roles of the different stakeholders were clearly defined.

### 3.2. Integration of Vaccination Within the School Environment

All four countries used schools as a key setting for reaching the target population, primarily between the ages of 10 and 13 years. However, the role assigned to schools varied considerably.

In Sweden and Finland, HPV vaccination was integrated into routine school health services. School health professionals directly managed a substantial part of the vaccination pathway, including providing information to families and students, obtaining or verifying consent, administering the vaccine, and ensuring follow-up.

In England, the model relied more heavily on specialised vaccination teams external to the school, through SAIS, while schools provided logistical and coordination support. Schools facilitated session planning, information dissemination, organisation of student flow, and communication with families. Australia represented an intermediate organisational model, combining professional vaccination services with strong school involvement in identifying eligible students, disseminating information, collecting consent, and engaging adolescents.

This comparison suggested that school-based vaccination did not necessarily require schools or school-based professionals to administer the vaccine themselves. However, all the models studied relied on close cooperation between the education system and healthcare professionals. Schools therefore represented an essential facilitating infrastructure, whether they hosted an external vaccination team or vaccination was integrated into established school health services.

### 3.3. Differentiated Consent Procedures Integrated into the Operational Vaccination Pathway

Consent procedures represented one of the main areas of variation across programmes. All countries provided for the information and involvement of parents or legal guardians, but approaches to obtaining consent and the extent to which adolescents were involved in decision-making differed.

In Australia and Sweden, parental consent was the usual approach, with paper-based or digital methods used depending on local arrangements. In Sweden, procedures could also vary between regions, particularly regarding the age or circumstances under which adolescents could participate in the decision themselves.

England had a particularly structured and graduated procedure: initial written consent, the possibility of seeking verbal consent when the consent form had not been returned, followed by an assessment of the young person’s capacity to provide consent independently according to the principle of Gillick competence. In Finland, assessment of the young person’s maturity and capacity to understand also played a central role, without a fixed age threshold.

Beyond these legal and cultural differences, an important finding concerned the need to integrate consent into an active follow-up process for unvaccinated students. The most structured approaches went beyond simply distributing a consent form: they included reminders, additional contact with families and, in some cases, an individual assessment of the adolescent’s capacity to consent. Consent therefore appeared not only as a legal requirement, but also as an organisational step that could directly influence the programme’s ability to reach its target population.

### 3.4. Multichannel Communication Tailored to Different Audiences

The programmes studied placed considerable emphasis on communication targeting both families and adolescents.

For families, multiple communication channels were used, including information letters or leaflets, consent forms, electronic communications, digital platforms, emails, text messages, reminders, and established school communication channels. Australia and England particularly illustrated this approach of active follow-up when consent documentation was not returned. In Finland, digital communication tools between schools and families could also be used by school health professionals.

Communication with adolescents represented a distinct component. In England, young people received specific information and were seen by the vaccination team, including when a consent form had not been returned. In Finland, information could be provided during school health consultations or classroom-based sessions.

These findings highlighted the value of a multilevel communication strategy that did not rely exclusively on providing information to parents. Adolescents were also active participants in the vaccination pathway, whose level of understanding and ability to ask questions should be taken into account.

### 3.5. Catch-Up as a Structural Component of the Programme

Another common feature of the programmes studied was the existence of mechanisms to vaccinate young people who missed the initial vaccination session. Approaches varied and included catch-up during a subsequent school-based session, vaccination through school health services, or referral to community clinics or primary care providers.

The English model particularly formalised this continuity by ensuring that young people who missed a school vaccination session remained identified and could be directed towards catch-up options. In Sweden, catch-up vaccination was available up to the age of 18 years within the programme, while in Finland, catch-up opportunities were provided within the school-based system.

In the programmes analysed, the vaccination session therefore did not constitute an isolated event. Rather, it formed part of a pathway that included identifying students who were absent or remained unvaccinated, following them up, and offering an alternative vaccination option. This organisational continuity appeared to be an important component in limiting missed opportunities for vaccination.

### 3.6. Information Systems Enabling Identification and Follow-Up of the Target Population

The more structured programmes also relied on systems for recording and monitoring vaccinations. Australia had a national immunisation information system. Sweden used a national register that enabled vaccination coverage to be monitored according to different criteria, including year of birth, sex, dose, and geographical area. In England, the Child Health Information Service contributed to identifying children and monitoring their vaccination status.

These systems made it possible to move beyond an approach focused solely on organising the school vaccination session. They enabled the eligible population to be identified, vaccinations administered to be documented, and children or adolescents who remained unvaccinated to be identified. They therefore provided potential support for individual follow-up, the organisation of catch-up vaccination, and programme evaluation.

### 3.7. Transferability and Implications for the French Context

The comparative analysis suggested that transferability should not be understood as the wholesale adoption of a national model. The programmes studied differed substantially in terms of healthcare system organisation, the role of school health services, the degree of decentralisation, and the legal framework governing consent. In contrast, the common organisational components appeared to be more transferable than the specific approaches used to implement them.

The potential transferability of the six common components was examined in relation to the current organisation of the French school-based HPV vaccination campaign. Table 2 summarises the international findings, the corresponding situation in France, the identified implementation gaps and the proposed adaptations, taking into account the French organisational, regulatory, educational, healthcare and sociocultural context.

## 4. Discussion

This comparative analysis of four international school-based HPV vaccination programmes identified six common organisational components: national leadership combined with structured local implementation; integration of vaccination within the school environment; an organised consent process; multichannel communication tailored to different audiences; structured catch-up mechanisms; and information systems enabling the identification and follow-up of the target population.

The four programmes examined in this study illustrate different organisational approaches to school-based HPV vaccination. Systems based on integrated school health services, such as those in Sweden and Finland, and systems relying on specialised vaccination teams, such as in England, were both represented among the selected programmes [16,17,18]. Across these different models, the comparative analysis identified organisational components covering successive stages of the vaccination pathway, from the identification of the target population through to the follow-up of unvaccinated adolescents. This finding suggests that the functions fulfilled by these components may be relevant across different organisational settings, although the comparison does not establish their individual contribution to vaccination coverage.

The relatively high HPV vaccination coverage reported in Australia and Sweden highlights the potential relevance of the organisational components identified in this study. Despite their different delivery models, both programmes illustrate the importance of coordination between health and education sectors, continuity of the vaccination pathway, and vaccination monitoring. These shared features offer organisational lessons for France, although the present analysis cannot determine their contribution to the coverage levels observed, which may also be influenced by contextual factors not assessed in this study. Placing these components in the context of the current organisation of the French campaign highlights both similarities with the international programmes studied and several areas that could be strengthened. In France, the national school-based vaccination campaign is already implemented within a structured interministerial framework involving health and education authorities at both national and regional levels and defining the main organisational arrangements for vaccination in schools [15]. However, the operational implementation of this framework relies on multiple stakeholders and may vary across regions and schools, particularly according to the availability of human resources, the vaccination services involved, and the capacity of school and healthcare teams to devote time to campaign preparation and follow-up [19]. One possible interpretation is that differences in how schools’ responsibilities are operationally defined may contribute to this variability. In some of the programmes studied, particularly in England and Australia, guidance more clearly specifies schools’ responsibilities before and during vaccination sessions [18,20]. Although our comparative analysis cannot establish whether more detailed guidance would reduce implementation variability in France, clarifying responsibilities at each stage of the campaign could be considered as a potential adaptation. These variations therefore do not necessarily reflect a lack of engagement among professionals, but may instead result from differences in available resources and in the operational framework supporting campaign implementation.

This issue is particularly important for the stages occurring before and after the vaccination session. Providing information materials and consent forms is a necessary component of the programme, but does not in itself ensure that the information is effectively received, understood, and acted upon by adolescents and their families. Differences in health literacy, language or digital barriers, and difficulties accessing school communication tools may limit the effectiveness of a uniform information strategy [13,14]. Our previous study conducted in the context of the French campaign highlighted difficulties associated with the use of digital consent procedures in some socially disadvantaged schools, leading several of them to maintain or reintroduce paper-based materials [19]. These findings suggest that, when considering adaptations to the French programme, particular attention should be paid to the accessibility of information and consent procedures for families facing linguistic, digital or other barriers. Free vaccination and school-based delivery may facilitate access, but additional measures may be needed to ensure that all families can engage with the vaccination pathway.

Socioeconomic inequalities may affect several stages of the school-based vaccination pathway, beyond access to information and consent procedures. Families facing financial, occupational or logistical constraints may have greater difficulty attending appointments outside school or accessing catch-up vaccination when an adolescent misses the scheduled session. Language barriers, health literacy and unequal access to digital tools may also affect engagement with the programme. Although the present comparison did not assess the impact of these factors on vaccination coverage, they should be considered when adapting the programme to the French context. Potential measures include maintaining accessible communication and consent options, identifying adolescents who miss school-based vaccination sessions, and facilitating catch-up opportunities that do not depend solely on families arranging vaccination independently. The feasibility and equity effects of these adaptations would require evaluation.

More broadly, the findings from the four programmes, considered alongside our previous observations in France, support examining school-based vaccination as a continuous pathway rather than as a series of isolated vaccination sessions. This pathway begins with the identification of the target population and includes providing information to adolescents and their families, obtaining consent, verifying vaccination status, administering the vaccine, recording the vaccination, and identifying and providing catch-up opportunities for adolescents who could not be vaccinated during the scheduled session. In our previous French study, missed opportunities for vaccination were observed despite favourable parental consent, particularly due to student absence, organisational constraints, or the unavailability of vaccination records [19]. Improvements in coordination and procedures during the second year of the campaign were accompanied by a reduction in missed opportunities, although these observations do not establish a causal relationship. Taken together, these findings provide a rationale for considering a pathway-based approach in France, with the aim of identifying and addressing potential disruptions at each stage of the vaccination process. The effectiveness of such an approach would need to be assessed through further evaluation.

The comparison of the four programmes suggests that the common organisational components may fulfil similar functions despite differences in their operational arrangements. This interpretation provides a basis for considering their potential adaptation to France, rather than replicating a foreign programme in its entirety. For example, clearly defined responsibility for organising and delivering vaccination is a common feature of the programmes studied, but this function may be fulfilled either by integrated school health services or by dedicated vaccination teams. Similarly, organising the collection and follow-up of consent represents a common function, whereas the approaches used to fulfil this function necessarily differ according to national legal, organisational, and sociocultural frameworks. As detailed in Table 2, the proposed adaptations for France were formulated by considering these common functions alongside the current French organisational and regulatory framework and the implementation gaps identified. These proposals should be regarded as context-informed recommendations, rather than interventions whose feasibility or effectiveness has been demonstrated by the present comparison.

This study has several strengths. Comparing programmes implemented across different healthcare, educational, and administrative contexts made it possible to identify common organisational components among the four programmes studied, beyond the specific characteristics of each national system. The complementary use of the scientific literature, institutional reports, international recommendations, and official national documents also enabled the inclusion of operational information that is often insufficiently detailed in scientific publications. Finally, the analysis went beyond a description of the programmes by seeking to distinguish the common functions fulfilled by their different components from their specific implementation approaches, providing a basis for considering their potential adaptation to the French context.

Several limitations should nevertheless be acknowledged. First, the purposive selection of only four countries does not constitute an exhaustive review of international school-based HPV vaccination programmes; consequently, some organisational approaches implemented in other contexts may not have been identified. The findings should therefore be interpreted as specific to the selected programmes and cannot be considered representative of international school-based HPV vaccination programmes as a whole. Second, the sources analysed were heterogeneous in terms of their nature, quality, and level of detail, and some operational characteristics may have been more extensively documented in certain countries than in others. As the documentary search was not systematic, relevant sources may have been missed, potentially affecting the completeness of the programme descriptions and the identification of common components. Furthermore, institutional documents primarily describe the intended organisation of programmes and may not capture the full range of variations occurring during local implementation. Third, the vaccination coverage estimates used to select the programmes referred to different years, age groups or birth cohorts, and vaccination endpoints. The absence of standardised, directly comparable coverage indicators precluded a formal comparison of programme performance. The comparative approach does not allow causal relationships to be established between the components identified and the observed vaccination coverage levels, which may be influenced by numerous contextual factors not examined in this study. As vaccination programmes and policies may evolve over time, the findings reflect the information available at the time of the documentary search, conducted between July and August 2026. Finally, the recommendations proposed for France are based on an analysis of transferability and have not yet been evaluated in terms of their feasibility, acceptability, or effectiveness under real-world conditions.

The recommendations arising from this analysis could help support the future development of the French school-based HPV vaccination programme. A next step could involve discussing the proposed components and adaptations with the main stakeholders concerned, including adolescents and families, healthcare professionals, schools, and institutions involved in organising the campaign, in order to assess their relevance, acceptability, and feasibility. This co-design process could lead to the development of a structured vaccination pathway adapted to the French context, specifying the different stages from the identification of eligible adolescents through to the follow-up and catch-up of those who remain unvaccinated, as well as the responsibilities of the different stakeholders at each stage. Particular attention should be paid to adapting this pathway to different local contexts and to populations that may face barriers to accessing information or vaccination. The adapted approaches could then be piloted and evaluated under real-world conditions, considering their feasibility, acceptability, equity, and ability to reduce missed opportunities for vaccination, as well as their impact on vaccination coverage. This approach would support the progressive development of the French programme based on available evidence, rather than considering its current organisation as a fixed model.

## 5. Conclusions

This comparative analysis highlights that, despite differences in healthcare and education systems, the school-based HPV vaccination programmes studied rely on a set of common components that contribute to structuring a coordinated vaccination pathway. The fact that these components are implemented differently across countries suggests that there is not necessarily a single model to replicate, but rather a set of essential functions whose implementation should be adapted to the organisational, regulatory, and sociocultural specificities of each context.

For France, the challenge may therefore be to strengthen a continuous vaccination pathway, from the identification and information of adolescents through to the follow-up and catch-up of those who remain unvaccinated, while seeking to minimise disruptions and inequalities in access at each stage. Our approach could thus help support the future development of the French school-based HPV vaccination programme, rather than considering the current model as fixed. Co-design and real-world evaluation of the proposed adaptations will be essential to determine their feasibility, acceptability, and contribution to improving vaccination coverage.

## Figures and Tables

**Table 1:** HPV vaccination coverage in the four selected countries.

Country	Reference Year	Age or Birth Cohort	Dose Definition	Girls (%)	Boys (%)	Source
Australia	2024	Aged 15 years	One dose	81.1	77.9	National HPV vaccination coverage data
England	2024–2025 academic year	Year 9 (13–14 years)	One dose	75.3	70.5	UKHSA
Finland	2025	Born in 2012	At least one dose	80	74	THL
Sweden	2025	Born in 2009	At least one dose	91.0	86.6	Folkhälsomyndigheten

**Table 2:** Transferability of the six common components of international school-based HPV vaccination programmes to the French context.

Common Component	International Findings	Current Situation in France	Identified Gap	Proposed Adaptation
1. National leadership and structured local implementation	National frameworks define programme objectives and responsibilities, while delivery is organised at territorial or institutional level.	An interministerial framework guides the national campaign, with implementation involving regional health authorities, vaccination services and schools.	Operational arrangements and the responsibilities of schools may vary across regions and establishments, depending partly on available resources.	Clarify the operational responsibilities of each stakeholder at every stage of the vaccination pathway, while allowing adaptation to local resources and organisational arrangements.
2. Integration of vaccination within the school environment	Vaccination is delivered in schools through integrated school health services or dedicated vaccination teams, with schools providing organisational and logistical support.	Vaccination sessions are delivered in collèges by external vaccination teams, in cooperation with schools. This model takes into account the limited human resources available within French school health services.	Coordination between schools and vaccination teams may vary, particularly during session preparation and follow-up, depending on local resources and organisational arrangements.	Strengthen coordination between schools and vaccination teams by defining their respective roles before, during and after vaccination sessions.
3. An organised consent process	All four programmes incorporate consent into the vaccination pathway, using procedures adapted to their legal frameworks. Some include reminders or additional contact with families when consent forms are not returned.	The French school-based campaign requires parental consent and uses consent forms distributed to families, with paper-based and digital arrangements depending on local implementation.	Distributing consent forms does not ensure that they are received, understood or returned. Digital procedures may also create access difficulties for some families.	Integrate consent into an active follow-up process, combining accessible paper-based and digital options with reminders and additional contact with families when forms are not returned, within the French legal framework.
4. Multichannel communication tailored to families and adolescents	The programmes use several communication channels to reach families and provide age-appropriate information to adolescents, with approaches adapted to local school and healthcare arrangements.	The French campaign provides information materials for families and adolescents, disseminated through schools and institutional communication channels.	A uniform communication approach may not reach all families equally, particularly where language, health literacy or digital access create barriers. Information provided to adolescents may also vary between schools.	Combine complementary communication channels and accessible formats for families with age-appropriate information and opportunities for discussion with adolescents, for example through classroom-based educational interventions, adapting delivery to the needs of each school community.
5. Structured catch-up mechanisms	The programmes provide alternative vaccination opportunities for adolescents who miss a school session, through subsequent school-based sessions, school health services or community-based providers.	The French campaign provides vaccination sessions in collèges, while vaccination is also available through community-based healthcare services.	Students who miss a school session may not be systematically identified and directed towards an alternative vaccination opportunity, creating missed opportunities despite favourable parental consent.	Establish a structured catch-up pathway to identify students who were not vaccinated during school sessions, inform families of available alternatives and facilitate access to vaccination through subsequent sessions or community-based services.
6. Information systems, monitoring and evaluation	Vaccination information systems support the identification of eligible adolescents, documentation of administered doses, monitoring of vaccination coverage and follow-up of those who remain unvaccinated.	Vaccinations administered during school-based sessions are recorded by vaccination services, while vaccinations delivered in community settings are documented through other healthcare and reimbursement systems.	Information available to individual stakeholders may not provide a complete and up-to-date picture of adolescents’ vaccination status across school-based and community settings. This may lead to missed opportunities, incomplete vaccination schedules or unnecessary additional doses.	Strengthen the availability and use of up-to-date vaccination information across school-based and community settings to support verification of vaccination status before administration, identify adolescents with incomplete vaccination schedules and facilitate appropriate follow-up, including targeted reminders to families by authorised organisations, in accordance with data protection requirements.

## Data Availability

All data analysed in this study were obtained from publicly available sources cited in the manuscript. The comparative data extracted for the four countries are provided in the Supplementary Materials.

## Supplementary Materials

The following supporting information can be downloaded at https://www.mdpi.com/article/10.3390/vaccines14100873/s1. Supplementary Table S1: Comparative matrix of school-based HPV vaccination programmes in Australia, England, Finland, and Sweden.

## Abbreviations

The following abbreviations are used in this manuscript:

GDPR	General Data Protection Regulation
HPV	Human Papillomavirus
SAIS	School-Aged Immunisation Services
WHO	World Health Organization
